# bra-miR9569 Targets the *BrAHA6* Gene to Negatively Regulate H^+^-ATPases, Affecting Pollen Fertility in Chinese Cabbage (*Brassica rapa* L. ssp. *pekinensis*)

**DOI:** 10.3390/plants14162604

**Published:** 2025-08-21

**Authors:** Siyu Xiong, Xiaochun Wei, Wenjing Zhang, Yanyan Zhao, Shuangjuan Yang, Henan Su, Baoming Tian, Fang Wei, Xiaowei Zhang, Yuxiang Yuan

**Affiliations:** 1 Institute of Vegetables, Henan Academy of Agricultural Sciences, Graduate T&R Base of Zhengzhou University, Zhengzhou 450002, Chinajweixiaochun@126.com (X.W.);; 2 School of Agricultural Sciences, Zhengzhou University, Zhengzhou 450001, China

**Keywords:** Chinese cabbage, Ogura CMS, bra-miR9569, *BrAHA6* gene, H^+^-ATPases

## Abstract

Ogura cytoplasmic male sterility (CMS) in Chinese cabbage (*Brassica rapa*) is characterized by complete pollen abortion, wherein stamens fail to produce viable pollen while pistils retain normal fertility. This maternally inherited trait is valuable for hybrid breeding. This study employed integrated analysis of miRNA, transcriptome, and degradome sequencing data aligned to the Chinese cabbage reference genome to elucidate the molecular function of bra-miR9569 in Ogura CMS pollen fertility and explore its associated pathways. Subsequently, a bra-miR9569 overexpression vector was constructed and transformed into *Arabidopsis thaliana*. Phenotypic characterization of transgenic Arabidopsis lines, combined with anther viability assessment and quantification of ATP content and reactive oxygen species (ROS) levels in Chinese cabbage, was performed to analyze the effects of bra-miR9569. Our findings demonstrate that mutation of the mitochondrial gene *orf138* in Ogura CMS lines leads to upregulation of bra-miR9569. This microRNA negatively regulates the expression of the ATP-related gene *AHA6*, resulting in reduced H^+^-ATPase activity. The consequent energy deficiency triggers cellular content degradation, ultimately causing failure of pollen wall formation and pollen abortion.

## 1. Introduction

Male sterility (MS) is a phenomenon in which the female gametes of a plant develop normally, and the male gametes develop abnormally but can receive external normal pollen for fertilization and fruiting [1]. There are two types of MS in plants: cytoplasmic male sterility (CMS) and genic male sterility (GMS). CMS is co-regulated by mitochondrial genes coupled to nuclear genes [2], and it commonly occurs in higher plants. In CMS plants, mitochondrial and nuclear genes interact to control male specificity, fertility development, and restoration in plants. CMS is maternally inherited and widespread [3]. Under this system, stamen morphology generally shows short filaments and tiny wrinkled anthers [4], but female functional organs are normal and can receive foreign pollen for fertilization to produce offspring.

Chinese cabbage is the most consumed cruciferous crop in East Asia, and it has been domesticated for about 6000 years; there are currently several varieties of Chinese cabbage, including spring and autumn types [5]. The leaves of Chinese cabbage are popular for their antioxidant capacity as well as their rich nutritional value, such as fiber and vitamins [6,7,8]. Cytoplasmic male sterile lines have become an important production pathway for utilizing the hybrid advantage in vegetables. Ogura-type cytoplasmic male sterility (Ogura CMS) has the advantages of complete pollen abortion, easy transfer, and progeny sterility up to 100%, which are widely utilized to breed cruciferous crops [9,10]. Currently, Ogura CMS is widely used in the crossbreeding of cruciferous vegetables, including Chinese cabbage [11], but the mechanism of its MS is still unclear. Currently, CMS has been reported in a variety of plants, and many mitochondrial genes responsible for CMS have been identified [12,13], which is significant for the in-depth study of the molecular mechanisms regulating CMS and the simplification of crop breeding for high-quality production.

MicroRNAs (miRNAs) are endogenous small RNAs that are about 18–24 nucleotides in length and that negatively regulate the expression of target genes at the transcriptional or post-transcriptional level by degrading target genes or inhibiting translation and DNA methylation of target genes [14]. miRNAs play an important role in biological processes, such as developmental regulation, and biotic and abiotic stress responses in plants [15]. Some miRNAs affect pollen development through post-transcriptional regulation [16,17,18], especially in the development of microspores [19] and the sporophyte chorioallantoic layer, a single-celled layer encasing the anther chamber that plays a key role in pollen development [20,21].

During plant growth and development, the pollen tube is the fastest growing plant cell, and it needs to consume a large amount of energy during polarized growth, as ATP plays an important role [22]. Studies have shown that the plasma membrane H^+^-ATPase (AHAs) in *Arabidopsis thaliana* is the largest family of cation-transporting P-type ATPases found in plants [23,24]. It is also involved in membrane potential formation, stress resistance, and cell signaling, which are essential for normal plant growth and development. The structure, function, and mechanism of action of H^+^-ATPases have been extensively studied [25,26], and H^+^-ATPases are believed to be key factors in the formation and maintenance of the transmembrane H^+^ gradient [27]. Pollen development and pollen tube growth are processes with high energy demands [28]. H^+^-ATPases are required for normal pollen germination and pollen tube elongation, and this AHA enzyme functions redundantly in pollen tubes [29]. In *Arabidopsis thaliana*, *AHA6*, *AHA8*, and *AHA9* are redundantly involved in the formation of the proton gradient and cell wall pattern in the pollen tube and are essential for polar growth of the pistil [30].

Mutations in the mitochondrial *orf138* gene trigger programmed cell death (PCD) in autogamous cells, leading to abnormal enlargement and content degradation, which results in vacuolization of the three-cell stage and pollen abortion [31]. In maize, by strongly interacting with *atp8* and *atp9*, the mitochondrial gene *atp6C* can disrupt the assembly of ATP synthase and reduce the activity of free hydrolase, leaving the demand for ATP unsatisfied during anther development, which increases reactive oxygen species (ROS) production, advances PCD in chorionic layer cells, and leads to pollen abortion [32]. In this study, we screened key differentially expressed miRNAs and target genes by sequencing the transcriptome, degradome, and miRNA library in Ogura CMS Chinese cabbage and maintainer lines with early and late pollen development and transformed *Arabidopsis thaliana* by constructing an OE-miR9569 overexpression vector. The mechanism by which bra-miR9569 is involved in CMS was further elaborated to provide new insights for hybrid seed production.

## 2. Results

### 2.1. Quality Control Analysis of Transcriptome Sequencing Data from Maintainer and Ogura CMS Chinese Cabbage Lines

**Figure 1:**
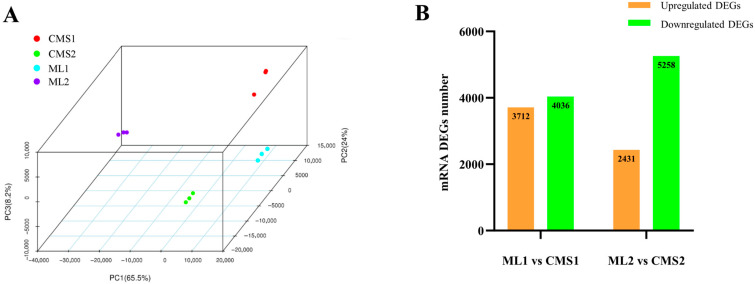
Sample principal component analysis and statistics of differentially expressed mRNAs. (**A**) Sample principal component analysis; (**B**) statistics of the number of differentially expressed genes in ML1 vs. CMS1 and ML2 vs. CMS2.

Illumina sequencing was performed at the early (buds 1.5–3.5 mm in length) and late (buds > 5 mm in length) stages of pollen development in the Chinese cabbage maintainer line (Y231-330) and the Ogura CMS line (Tyms), with three biological replicates for each period. After sequencing quality control, 138.12 GB of clean data were retained, of which, ML1 (sample IDs: S07, S08, S09), the Chinese cabbage maintainer line at the early stage of pollen development, had an average of 77,421,534 clean reads; and ML2 (sample IDs: S01, S02, S03), at the late stage of pollen development, had an average of 78,679,234 clean reads. The Ogura CMS line had an average of 76,235,037 clean reads in the early stage of pollen development (CMS1, sample IDs: S10, S11, S12) and 75,917,886 clean reads in the late stage of pollen development (CMS2, sample IDs: S04, S05, S06). The GC content of the 12 samples ranged from 43.46 to 45.57%, and Q30 was greater than 95.5% for all samples. The minimum number of mapped reads localized to the reference genome of Chinese cabbage version 1.5 was 55,082,768, and the maximum number of mapped reads was 70,645,510, accounting for 72.23–88.59% of the clean reads (Table 1). These results showed that the sequencing data were of good quality and reliable and could be uniquely compared to the reference genome of Chinese cabbage. Three-dimensional principal component analysis (PCA) showed that the components were independent of each other, and the biological replicates were tightly clustered, indicating a high degree of independence between the samples and a strong correlation between the three replicates (Figure 1A). Using a false discovery rate (FDR) of 0.01 and a fold change criterion (Fold Change) of 2, the screen identified the presence of 3712 upregulated differentially expressed genes (DEGs) and 4036 downregulated DEGs in the early stages of pollen development in both lines. The number of up- and down-regulated DEGs identified in the late stages of pollen development in both lines were 2431 and 5258, respectively (Figure 1B).

The number of single nucleotide polymorphism (SNP) loci, proportion of transformation types, proportion of subversion types, and proportion of heterozygous SNP loci screened for each sample were counted (Table 2). The number of SNP loci was slightly higher during early pollen development than during late pollen development in both the maintainer line (Y231-330) and the Ogura CMS line (Tyms), and most of the SNP loci detected were located in the gene coding region. The percentages of the total number of SNP sites detected in the 12 samples were 57.37, 42.63, and 35.46% for conversion-, subversion-, and heterozygous SNP sites, respectively. CMS1 and CMS2 refer to the early and late stages of pollen development in sterile lines, while ML1 and ML2 refer to the early and late stages of pollen development in maintainer lines.

### 2.2. Quality Control of miRNA Sequencing Data from Anther Samples of Both Lines

To study the regulation of miRNA on the fertility of Ogura CMS, we constructed small RNA sequencing libraries from anthers of sterile and homozygous maintainer lines at different stages of pollen development and set up three biological replicates at two different stages for the two lines. Small RNA sequencing of 12 samples was completed, and 143.64 Mb of clean reads were obtained, with no less than 10.04 Mb of clean reads for each sample. The base quality values of Q30 were more than 96.11%, indicating that the sequencing data were of high quality and reliable (Table 3). After filtering the small RNA sequences without reads, the length distribution of small RNAs was 18–24 nt (Supplementary Material SA Figure S1). Most of the RNAs had sequence sizes of 19–24 nt, with most small RNAs in the library having 21 nt, followed by small RNAs of 24 nt in length.

**Figure 2:**
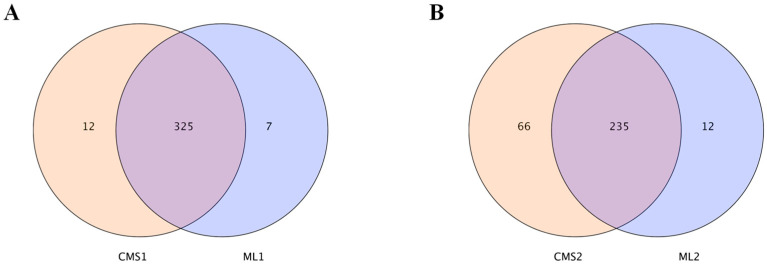
Statistics of the number of differential miRNAs at two periods of pollen development in the Ogura CMS line and maintainer line. (**A**) Number of differential miRNAs at the early stage of pollen development in maintainer and sterile lines; (**B**) number of differential miRNAs at the late stage of pollen development in maintainer and sterile lines.

miRNA sequencing data were statistically analyzed. The two lines had 325 commonly expressed miRNAs during early pollen development, with 12 miRNAs specifically expressed in the sterile line and only 7 miRNAs specifically expressed in the maintainer line (Figure 2A), whereas 235 commonly expressed miRNAs were found during late pollen development, with 66 miRNAs specifically expressed in the sterile line and 12 miRNAs specifically expressed in the maintainer line (Figure 2B).

### 2.3. Sequencing Analysis of the Degradome of Two Chinese Cabbage Lines

To identify the target genes of miRNAs obtained using miRNA sequencing, anther samples from different pollen development periods of the Ogura CMS line and its maintainer line were mixed, and a degradome library was constructed for late sequencing analysis. A total of 19.00 M clean tags were obtained by sequencing. The original tags obtained from sequencing, clean tags and cluster tags, were obtained after quality inspection, both of which were 47 nt in length, and a total of 18,995,289 clean data were obtained. The sequencing data from the samples were compared to the reference genome of Chinese cabbage (*Brassica rapa* v1.5). The comparison results are shown in Table 4. The *p*-value was set at less than 0.05, and miR9569 was detected to shear the target gene Bra013168 (*AHA6*) at locus 582 (Supplementary Material SA Figure S2).

### 2.4. Joint Analysis of miRNAs and Their Target Genes by miRNAomics

Differentially expressed miRNAs from miRNA and degradome sequencing of anthers of the Ogura CMS line and its isoforms at different pollen developmental stages were jointly analyzed with DEGs identified in transcriptome sequencing. The 22 genes regulated by novel_mir_403, novel_mir_448, novel_mir_51, and novel_mir_95 were differentially expressed miRNAs and DEGs (Supplementary Material SB). The NR annotations of these genes were sucrose transporter proteins, growth hormone response factors, cytochrome c biosynthesis proteins, cysteine proteases, bet v I allergenic family proteins, MYB-associated proteins, adenosine triphosphate conjugating, and H^+^-ATPase.

### 2.5. H^+^-ATPases Related Genes Are Significantly Downregulated During Anther Development in the Ogura CMS Sterile Line of Chinese Cabbage

**Figure 3:**
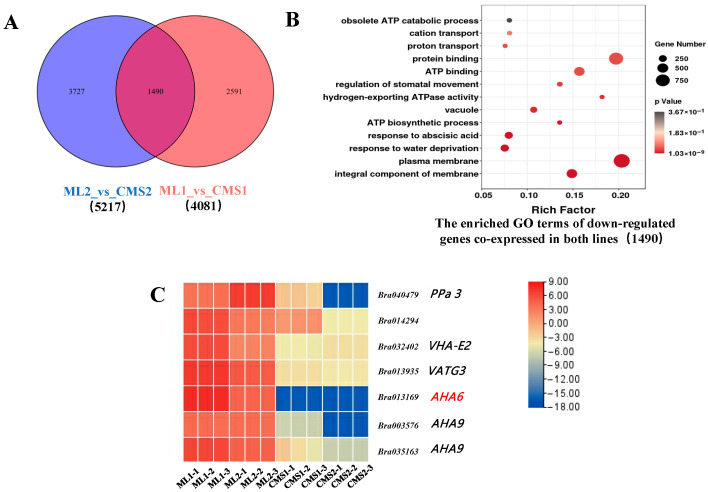
Intergroup comparison of downregulated differentially expressed genes of the Ogura CMS and maintainer lines. (**A**) Number of differentially expressed and commonly expressed genes between the two groups; (**B**) GO enrichment analysis of downregulated commonly expressed genes between the two groups; (**C**) heatmap of H^+^-ATPases differentially expressed genes in the Ogura CMS and maintainer lines of Chinese cabbage.

This study utilized sequencing data from the miRNA, transcriptome, and degradome libraries of the Ogura CMS line and its isoform maintainer line (NCBI; CNGBdb). Screening statistics of genes downregulated in the maintainer and sterile lines during both periods revealed 1490 commonly expressed DEGs, with 3727 DEGs specifically expressed during the early stage of pollen development and 2591 genes expressed during the late stage of pollen development in both lines (Figure 3A). GO enrichment analysis of differential mRNAs commonly expressed in sterile and maintainer lines showed that they were mainly enriched in the entries of plasma membrane, proton translocation, and H^+^-ATPase activity (Figure 3B). Visualization of ATP-related gene expression in a heat map revealed that gene *Bra013168* (*AHA6*) was significantly downregulated during pollen development in the sterile line (Figure 3C). *AHA6* is an H^+^-ATPase that may be involved in energy metabolism, thereby affecting the formation of the pollen wall during pollen development and leading to pollen abortion. *PPa3* is a gene that encodes a protein with inorganic pyrophosphorylase activity that promotes the hydrolysis of pyrophosphate to phosphoric acid, thereby participating in ATP synthesis. VHA-E2 is an H^+^-ATPase subunit, and VTAG3 is a V-ATPase G-subunit-like protein. *AHA6* and *AHA9* are both H^+^-ATPases. VHA-E2, VTAG3, *AHA6*, and *AHA9* affect ATP generation by the binding of adenosine diphosphate to phosphoric acid, suggesting that a certain regulatory relationship between ATP and pollen septation may exist.

We preliminarily hypothesized the pathway mechanism of pollen abortion caused by the mutation of the mitochondrial gene *orf138*. The mutation of the mitochondrial gene *orf138* in the Ogura CMS line caused the downregulation of ATP related genes, triggering the reduction of H^+^-ATPases and the lack of energy, which led to content degradation, failure to form the pollen wall, and pollen abortion.

### 2.6. Analysis of bra-miR9569 Targeting Relationship with Target Genes and Bioinformatics Analysis

To identify the targeting relationship between miR9569 and its target gene *AHA6*, this study analyzed the target location between miR9569 and its target gene *AHA6* using psRNATarget and psRobot software and found that miR9569 cleaves to the target gene *AHA6* at 582 bp of its sequence (Supplementary Material SD Figure S1). This anchors the targeting relationship between the two. Mature and precursor sequences of miR9569 were obtained from the miRBase database (Supplementary Material SC Table S1). Chromosomal localization of the miR9569 precursor and its target gene *AHA6* revealed that both were located on chromosome A03. The results for the amplification of miR9569 precursor gene sequences are shown in Supplementary Material SD Figures S2 and S3. The secondary structure of the miR9569 precursor was predicted using RNAfold, and the red line shows the location of the sequence of its mature body (Supplementary Material SD Figure S4). Base conservation of the miR9569 mature body sequence was analyzed based on the software Weblogo and showed that miR9569 was highly conserved at bases 7, 9, 10, 12, and 15 (Supplementary Material SD Figure S5).

### 2.7. qRT-PCR Validation of miR9569/AHA6 Regulation and Differential Expression Profile Reliability

**Figure 4:**
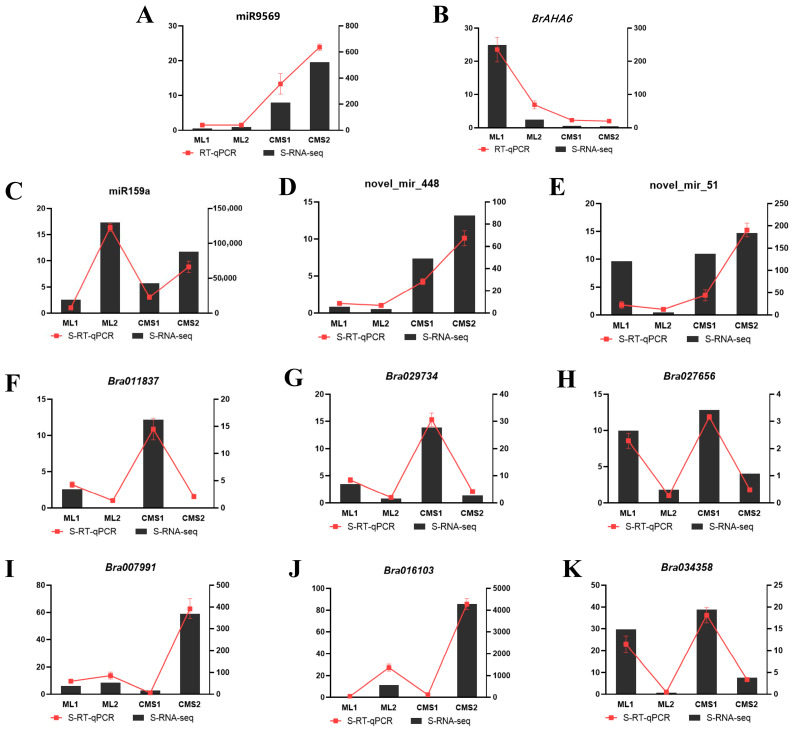
qRT-PCR validation of pollen fertility-related miRNAs and mRNAs. (**A**,**B**) qRT-PCR validation of bra-miR9569 and its target gene, *BrAHA6*. (**C**–**E**) qRT-PCR validation of known miRNA (miR159a) and novel miRNAs (novel_mir_448, novel_mir_51). (**F**–**K**) qRT-PCR verification of six differentially expressed genes related to ATP pathway.

miR9569 and its target gene, *AHA6*, were selected at different developmental periods in the Ogura CMS and maintainer lines and subjected to quantitative fluorescence analysis to verify the negative regulatory relationship between miRNAs and target genes. miR9569 expression increased significantly with pollen development in the Ogura CMS line, while the expression was lower in the maintainer line. The expression of its target gene, *AHA6*, was higher at the early stage of pollen development in the maintainer lines, and the expression of its target gene decreased with pollen development. It was not expressed in the sterile line. The fluorescence quantification results revealed that miR9569 negatively regulated the target gene *AHA6* (Figure 4A,B).

One known miRNA (miR159a) and two new miRNAs (novel_miR_448, novel_miR_51) that were differentially expressed and six DEGs were selected to validate the reliability of the transcriptome sequencing data. The qRT-PCR measurements of these miRNAs (Figure 4C–E) and DEGs (Figure 4F–K) showed consistent expression trends with the sequencing FPKM (Fragments Per Kilobase of exon model per Million mapped fragments) values. These results showed that the selected and sequenced results had the same trend, demonstrating the reliability of the sequencing data.

### 2.8. Genetic Transformation of OE-miR9569 Arabidopsis Thaliana and Identification of Positive Seedlings

In this study, homologous recombination and golden gate seamless cloning were used for vector construction, and the recombinant plasmid OE-miR9569 was digested using EcoRV endonuclease. The recombinant plasmid fragments were of the correct size (Supplementary Material SE Table S1). The structure of the OE-miR9569 vector is shown in Supplementary Material SE Table S2. The constructed vector was subjected to agarose gel electrophoresis by colony PCR (Supplementary Material SE Table S3). Positive colony PCR products were sequenced, and the sequencing results were correct, indicating that the OE-miR9569 vector was successfully constructed.

**Figure 5:**
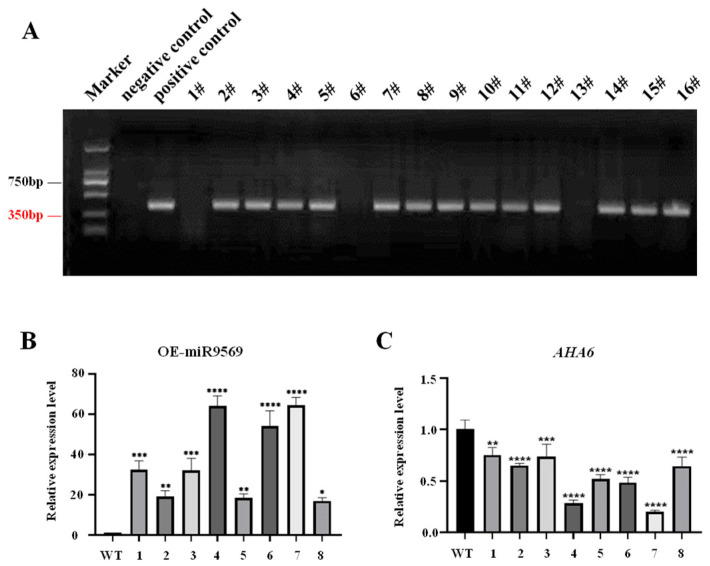
Identification results of OE-miR9569-positive plants. (**A**) Identification of eukaryotic resistant antibiotics in the T1 generation of OE-miR9569. (**B**) Fluorescence quantification of miR9569 expression in the T2 generation of OE-miR9569 plants. (**C**) Fluorescence quantification of *AHA6* expression in the target gene in the T2 generation of OE-miR9569 plants. One-way ANOVA using multiple tests (* means *p* < 0.05; ** means *p* < 0.01; *** means *p* < 0.001; **** means *p* < 0.0001).

The OE-miR9569 transgenic *Arabidopsis* seeds obtained by the flower dipping method were sown in 1/2MS medium containing genistein (G418). After about six weeks, DNA was extracted, and the antibiotic sequence fragment was amplified by PCR for the preliminary verification of positive seedlings. The presence of bands revealed that the vector was successfully transferred into plants (Figure 5A). After preliminary screening, RNA was extracted from the plants, and after designing primers for miR9569 expression detection, miR9569 and its target gene *AHA6* were detected by fluorescence quantification. Compared with the wild-type plants, miR9569 expression in the OE-miR9569 transgenic plants was significantly increased (Figure 5B), and the expression of its target gene was significantly decreased (Figure 5C). Plants with significant changes in the expression of miR9569 and its target gene were selected. OE-miR9569-positive transgenic *Arabidopsis* plants were obtained, and the seeds were harvested and preserved after the pods matured.

### 2.9. Phenotypic Analysis Reveals miR9569 Regulates Pollen Dispersal and Maturation in Arabidopsis

**Figure 6:**
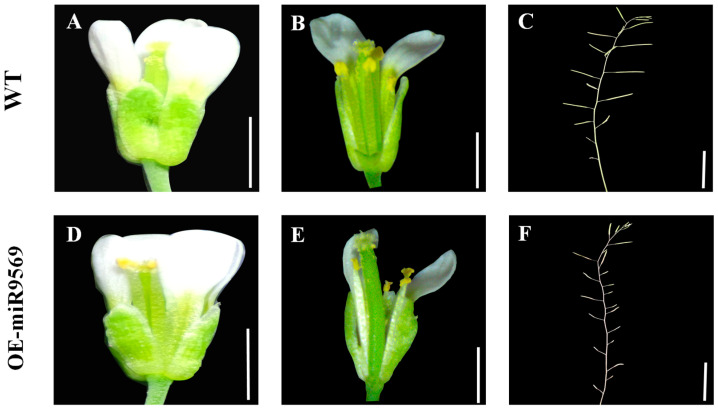
Observation of phenotypic characteristics of wild-type and OE-miR9569 mutant *Arabidopsis thaliana*. (**A**,**D**) Observation of wild-type and miR9569 mutant inflorescences. (**B**,**E**) Observation of anthers and stigmas at anthesis of wild-type and miR9569 mutant. (**C**,**F**) Observation of wild-type and miR9569 mutant siliques. Scale bar = 0.5 mm (**A**,**D**), 0.4 mm (**B**,**E**), 1 cm (**C**,**F**).

To verify the function of miR9569, the OE-miR9569 *Arabidopsis* mutant and the wild type were phenotypically characterized in this study. There were no significant differences in inflorescence morphology size between wild-type *Arabidopsis* and OE-miR9569 transgenic *Arabidopsis* (Figure 6A,D). The anthers were then observed after removing their petals with forceps, and the amount of pollen dispersed by OE-miR9569 transgenic *Arabidopsis thaliana* was significantly reduced compared to that dispersed by the wild type during the flowering stage (Figure 6B,E). Observation of siliques at maturity in both materials revealed that OE-miR9569 transgenic *Arabidopsis thaliana* had smaller pods that were shriveled and not full compared to the wild type (Figure 6C,F). These studies indicate that miR9569 affects plant pollen fertility to a certain extent.

### 2.10. miR9569 Overexpression Compromises Pollen Viability and Anther ATP Levels via H^+^-ATPase Dysregulation

**Figure 7:**
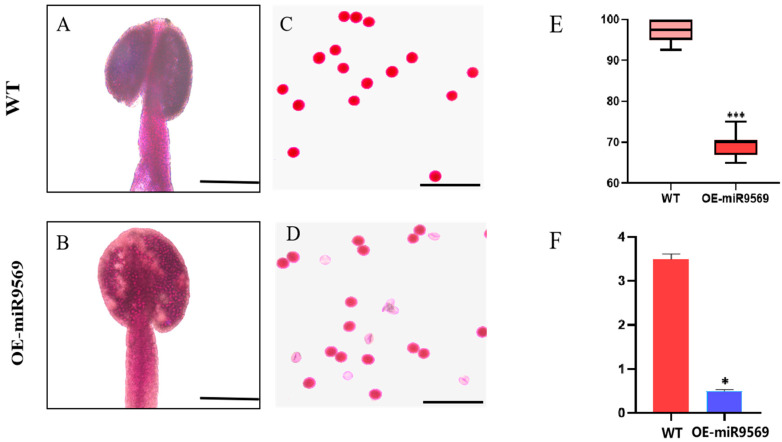
Identification of pollen viability and determination of ATP content in wild-type and OE-miR9569 mutants. (**A**,**B**) Alexander rectification staining of anthers, scale bar = 200 μm. (**C**,**D**) Alexander staining of pollen grains, scale bar = 100 μm. (**E**) Stainability statistics (%). (**F**) ATP content determination (µmol/g). One-way ANOVA (* means *p* < 0.05; *** means *p* < 0.001).

To further analyze the differences between OE-miR9569 transgenic *Arabidopsis thaliana* and the wild type, this study observed anther staining and identified the pollen viability of the OE-miR9569 *Arabidopsis thaliana* mutant and wild-type *Arabidopsis thaliana*. OE-miR9569 transgenic *Arabidopsis thaliana* had a certain degree of pollen inactivity compared with the wild type, and the whole stained anthers were transparent (Figure 7A,B). The inactivated part of pollen grains was not stained a purple–red color (Figure 7C,D), and the rate of pollen grain stainability was significantly reduced (Figure 7E). The ATP content of the anthers of both materials was measured, and it was found that the ATP content of the anthers of OE-miR9569 transgenic *Arabidopsis thaliana* was significantly decreased compared with that of the wild type (Figure 7F).

These findings suggest that miR9569 may affect pollen fertility by influencing H^+^-ATPases and that upregulated expression of miR9569 causes pollen inactivity or failure to form normal full oval pollen grains, reducing plant fertility.

### 2.11. Anther Cavitation and ROS Toxicity: Key Phenotypes of Pollen Sterility in miR9569-Overexpressing Arabidopsis

**Figure 8:**
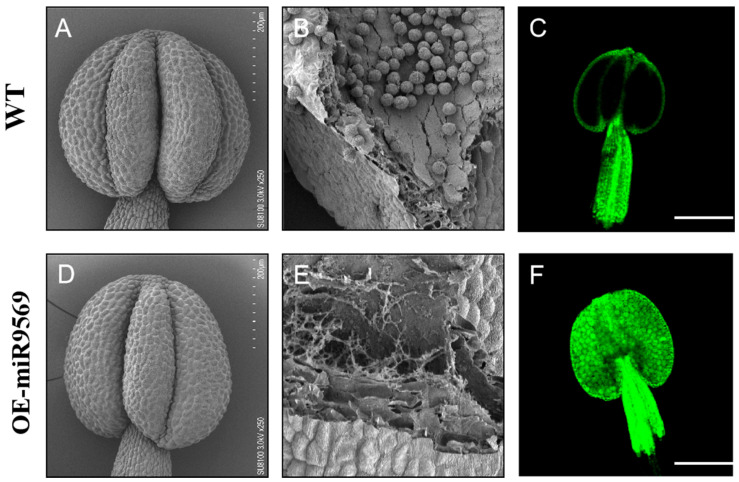
Scanning electron microscopy observation of the ultrastructure and identification of reactive oxygen species in wild-type and OE-miR9569 anthers. (**A**,**D**) Scanning electron microscopy observation of wild-type and OE-miR9569 anthers at the unopened stage. (**B**) Wild-type *Arabidopsis* anthers with normal elliptical full pollen grains. (**E**) Partial local magnification of the mature anthers of OE-miR9569 at the unopened stage, which appeared to be empty chambers. Scale bar = 200 μm (**A**,**D**), 50 μm (**B**,**E**). (**C**,**F**) Confocal microscopy observation of reactive oxygen species staining results; scale bar = 200 μm.

To observe the anthers and their internal structural status between the OE-miR9569 mutant and wild-type *Arabidopsis thaliana*, a scanning electron microscope was used to observe the anthers and pollen grains inside the anthers. There were no significant differences between their anthers at the unflowering stage (Figure 8A,D). Local magnification of the anthers revealed that wild-type *Arabidopsis* anthers were filled with a large number of full pollen grains (Figure 8B), whereas the local magnification of the internal parts of the OE-miR9569 transgenic *Arabidopsis* anthers showed empty chambers with few viable pollen grains (Figure 8E). The interior of wild-type *Arabidopsis* anthers was largely unstained, indicating that both had less internal ROS accumulation (Figure 8C), whereas the interior of OE-miR9569 transgenic *Arabidopsis* anthers was filled with green fluorescence, indicating that their anthers had more internal ROS accumulation (Figure 8F).

In summary, OE-miR9569 transgenic *Arabidopsis thaliana* has almost no production of normal pollen grains in some areas inside the anthers. Thus, it is hypothesized that the toxic effect of the large ROS accumulation inside its anthers reduces pollen fertility.

## 3. Discussion

In this study, we obtained high-quality sequencing results by sequencing the sRNA, degradome, and transcriptome of the Ogura CMS line and its homozygous maintainer line of Chinese cabbage. The transcriptome data were statistically analyzed, and the genes downregulated in the two periods in both maintainer and sterile lines were screened, revealing 1490 DEGs. GO enrichment analysis of these DEGs showed that they were mainly enriched in the plasma membrane, proton transport, and H^+^-ATPase activity, indicating that pollen development was closely related to H^+^-ATPases. Starch metabolism provides energy and a carbon skeleton for pollen development, germination, and pollen tube elongation and is decisive for pollen biological function and fertility. In barley, ATP-driven starch synthesis fuels pollen maturation [33,34], with ATP availability being rate-limiting for starch accumulation. Chili pepper CMS lines exhibit premature ATP hydrolysis during the tetrad stage, followed by undetectable H^+^-ATPase activity in uninucleate microspores, directly causing male sterility [35]. ATP is one of the main factors affecting pollen development and is crucial for pollen development, with large amounts of energy required for processes such as timely PCD of chorioallantoic cells and pollen wall formation [29].

In plant cells, mitochondria are important sites for ATP synthesis, and CMS expression is the result of subtle interactions between mitochondrial and nuclear genes [36]. Arabidopsis plasma membrane H^+^-ATPases (AHAs; notably AHA6/8/9) establish proton gradients that direct pollen tube tip growth through cell wall remodeling [30]. In maize, the mitochondrial gene *atp6C* is unable to meet the ATP requirement during anther development by interacting with *atp8* and *atp9*, leading to pollen abortion [32]. We previously identified Brassica-specific miR9569 as a sterility-linked regulator that targets pollen-essential genes through its anther-specific overexpression in CMS lines [11]. Genome-wide analysis of the transporter protein genes expressed in the four developmental stages of the male gametophyte has shown that *AHA6* is specifically or preferentially expressed relative to the sporophyte tissue [37]. The absence of AHAs leads to a decrease in energy, thus affecting pollen development [30], which is consistent with the results of the present experimental study.

**Figure 9:**
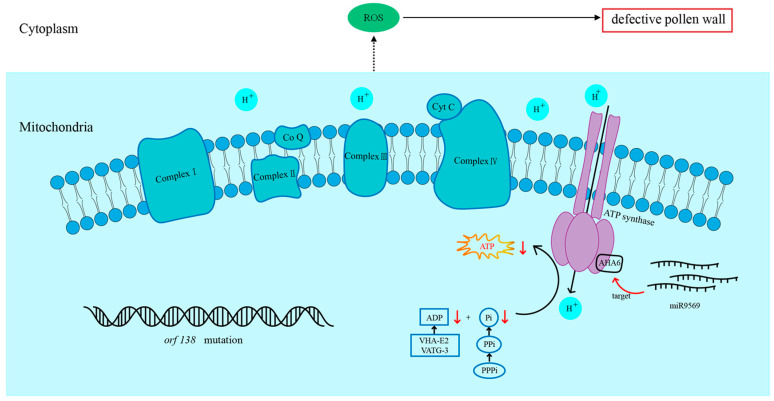
The miR9569 target, the *BrAHA6* gene, regulates the pollen fertility pathway in Chinese cabbage.

This study established a mechanistic framework (Figure 9) in which the specific overexpression of miR9569 in anthers of the Chinese cabbage Ogura CMS line inhibits *BrAHA6*, a plasma membrane H^+^-ATPase that is crucial for pollen development. Key research findings indicate that miR9569-mediated downregulation of *BrAHA6* disrupts the H^+^ electrochemical gradient across the plasma membrane, and H^+^-ATPase is a key factor in the formation and maintenance of the transmembrane H^+^ gradient [27]. The aberrant H^+^ gradient compromises secondary transport systems, ultimately affecting cellular energy homeostasis. Elevated ROS and ATP levels observed in miR9569-overexpressing (OE) lines reflect compensatory metabolic stress or failed energy utilization downstream of H^+^-ATPase dysfunction. Impaired proton pumping hinders ATP-dependent processes that are critical for fertility, impeding pollen wall formation and timely PCD of chorionic cells. Consequently, *BrAHA6* suppression by miR9569 disrupts pollen wall patterning and tapetal PCD, directly leading to reduced pollen fertility. These findings underscore *BrAHA6* as a pivotal node linking miRNA regulation to energy metabolism and structural development in pollen. In conclusion, this study provides theoretical guidance for the genetic breeding of Chinese cabbage through the study of the effect of miR9569 on pollen fertility.

## 4. Materials and Methods

### 4.1. Experimental Materials

The Ogura CMS Chinese cabbage line (Tyms) and its maintainer line (Y231-330) selected for this study were provided by the Institute of Vegetables, Henan Academy of Agricultural Sciences (Zhengzhou, China), and were planted in Henan Modern Agricultural Research and Development Base (Yuanyang, China) [11]. Seeds were selected and sown in seed trays filled with substrate. During the germination period, the temperature was maintained at 25 °C. After transplanting to the field, timely control measures were taken against pests, diseases, and weeds to avoid the impact of non-natural factors on the experiment. Regular irrigation was carried out, and observations were made.

### 4.2. RNA Extraction and Library Construction

Total RNA was extracted from anther samples by using RNAiso Plus (Takara BioTechnology Co., Ltd., Dalian, China). The purity and concentration of total RNA were determined using a micro-volume spectrophotometer (Nano-500), and RNA integrity was assessed by 1.2% agarose gel electrophoresis. Total RNA samples that passed the test were separated into fragments using polyacrylamide gel electrophoresis, and small RNAs in the range of 18–30 nt were recovered using gel recovery. The small RNA libraries were obtained using reverse transcriptase, and libraries that passed the quality test were used in high-throughput sequencing with the HiSeq X-ten platform (Biomark Biotechnology Co., Ltd., Beijing, China). Raw reads were processed according to the base quality value [38] and nucleotide number to obtain high-quality sequences (clean reads). Bowtie software [39] was used to compare and process the clean reads to obtain the unannotated reads (including miRNAs), and sequence matching was performed with the corresponding reference genomes.

### 4.3. Construction of the cDNA Library and Data Processing

Sequencing of cDNA libraries using sequencing by synthesis (SBS) technology generates a large amount of high-quality data, which is referred to as raw data. The raw data generated in FASTQ format must be cleaned to remove low-quality reads and those containing adapters and poly-N sequences, resulting in clean reads. Clean reads were aligned against the Chinese cabbage reference genome v1.5 (http://39.100.233.196:82/download_genome/) (29 July 2025). The StringTie (1.3.1) software was used to calculate the FPKM values for mRNA and coding genes in each sample. The Bioconductor package EdgeR (1.10.1) [40] was used to identify differentially expressed genes, while Poisson distribution and empirical Bayesian methods were employed to reduce the coefficient of variation among transcripts, thereby enhancing the reliability of the analysis results. The screening criteria were a fold change (FC) ≥ 2 and a false discovery rate (FDR) < 0.05. All analyses were performed using software tools on the BMK Cloud platform (https://www.biocloud.net/) (29 July 2025).

### 4.4. Construction of the Degradome Library and Statistics for the Sequencing Results

Total RNA was extracted at different pollen development periods in Ogura CMS and maintainer Chinese cabbage lines and mixed into one sample in equal amounts to construct the degradome library. Clustering data were compared with the Rfam database [41] to obtain relevant annotation information, and the unannotated sequences were subjected to subsequent degradation site analysis. Degradation sites were detected by Cleaveland [42] software using the known miRNAs, miRNAs predicted from the small RNA analysis, and the sequence information of the transcripts of the corresponding species genes (Y231-330 and Tyms).

### 4.5. qRT-PCR Validation of miRNA and mRNA Sequencing Data

The target miRNAs were subjected to real-time fluorescence quantitative PCR (qRT-PCR) by adding poly-A at the 3′ end to verify the reliability of the miRNA sequencing data. miRNA reverse transcription was performed using a Takara Mir-XmiRNA First-Strand Synthesis Kit (Takara BioTechnology Co., Ltd., Dalian, China) and TB GreenTM qRT-PCR User Manual Reverse Transcription Kit (Takara BioTechnology Co., Ltd., Dalian, China). mRNA reverse transcription and qRT-PCR were performed using kits from Takara. Based on the mRNA sequences, Primer 5.0 was used to design the primers for mRNA fluorescence quantitative PCR. GAPDH screened in our laboratory was used as the internal reference primer, and primer synthesis was performed by Zhengzhou Sunya Biotechnology Co. (Zhengzhou, China). All qPT-PCR experiments were performed on the LightCycler^®^ 480 Instrument II (F. Hoffmann-La Roche, Basel, Switzerland), and the expression levels of relevant genes for -miRNA and mRNA were analyzed using the 2^−ΔΔCt^ method [43]. qRT-PCR-related primer sequences are listed in Supplementary Material SC Tables S2 and S3.

### 4.6. OE-miR9569 Arabidopsis Thaliana Transformation

The genetic transformation material used in this study was *Arabidopsis thaliana* (Columbia, 2n), provided by the Institute of Vegetables, Henan Academy of Agricultural Sciences (Zhengzhou, China). *Agrobacterium tumefaciens* (GV3101) and *Escherichia coli* (DH5α) were used as sensory cells by Weidi Biological Company (Shanghai, China); the vector interference and overexpression vectors used were the pBWA(V)KS-ccDB vector provided by Biorun Biological Company (Wuhan, China).

*Arabidopsis thaliana* was sown and incubated in a light incubator with 16 h of light (22 °C) and 8 h of dark (18 °C). The culture was then expanded using the flower dipping method with *Agrobacterium tumefaciens* and activated by shaking at 28 °C for 2 days. *Arabidopsis thaliana* was then infested by the flower dipping method. *Agrobacterium* spp. was expanded and activated by shaking at 28 °C for 2 days, transferred to centrifuge tubes, centrifuged at 6000 rpm for 10 min, and resuspended in osmotic solution (containing 5% sucrose, 0.02% SilwetL-77, and 1/4 MS (Murashige and Skoog) nutrient solution with Acetosyringone) to form *Agrobacterium* spp. *Agrobacterium tumefaciens* was resuspended in an osmotic solution (OD600 = 0.8). The siliques and open flowers of 6-week-old *Arabidopsis* plants were removed, and the unopened flower buds were removed with forceps. The *Arabidopsis* seedlings were inverted, and their inflorescences were immersed in the prepared suspension for 20–30 s to fully infest the inflorescences. After infestation, an appropriate amount of water was sprayed onto infested *Arabidopsis*, and the infested inflorescences were covered with black plastic film and grown in a dark box for 24 h. The treated *Arabidopsis* plants were placed in a light incubator (16 h of light at 22 °C, 8 h of dark at 18 °C) for normal growth. The infested inflorescences were re-infested after one week, and the infested inflorescences were harvested at maturity after about three weeks.

### 4.7. Identification of Pollen Viability

Plant pollen grain viability was determined by Alexander staining. Viable pollen grains stained with Alexander’s stain were a distinct purple–red color, while pollen that was not stained with Alexander’s stain had abnormal coloring. Alexander staining was performed using an appropriate amount of Alexander staining solution placed in a 200 µL EP tube. The mature anthers were removed with tweezers under the stereo microscope (Toup Optoelectronics Technology Co., Ltd., Hangzhou, China) and placed into the centrifuge tube containing the Alexander staining solution. They were stained for 1 h under light protection, and the stained anthers were rinsed with water. They were placed on slides (with an appropriate amount of water added to the surface), adjusted, and covered with a coverslip. The slides were then observed, and images were acquired after microscopic examination of the film.

### 4.8. Determination of the ATP Content and ROS Content

We selected *Arabidopsis thaliana* in full bloom as the experimental material. The ATP content of the anthers of transgenic *Arabidopsis thaliana* was determined using a reagent kit (Grace Biotechnology Co., Suzhou, China). To determine the active oxygen content, the floral organs, including petals, calyx, and stigma, of transgenic *Arabidopsis thaliana* were removed with tweezers, and the anthers were placed in 1.5 mL EP tubes with freshly prepared MES-KCl buffer. The material was completely immersed, and the liquid in the centrifuge tubes was carefully removed with a syringe after continuous immersion for 30 min. The dye H2DCFDA was added to the centrifuge tubes for a final concentration of 50 µM for 1 h. The dye was aspirated with a syringe. The samples were rinsed two or three times with MES-KCl buffer solution and soaked in MES-KCl buffer for 15 min. The samples were removed and placed on slides, prepared (not pressed) with a drop of MES-KCl buffer, and then viewed and photographed using the FITC channel of a confocal microscope (LSM880, ZEISS).

## 5. Conclusions

In this study, we sequenced the Ogura CMS and maintainer lines of Chinese cabbage at the early and late stages of pollen development. After quality control analysis, the sequencing data of miRNAs, transcriptomes, and degradomes were highly reliable and verified to be consistent with the sequencing results obtained by qRT-PCR. Through gene differential expression, target prediction, and functional enrichment analysis, we identified a regulatory relationship between miR9569 and the differentially expressed gene AHA6, and constructed a miR9569 overexpression vector, which was then transformed into *Arabidopsis thaliana*. Comparative analysis of wild-type and OE-miR9569 transgenic *Arabidopsis thaliana* was performed by phenotypic observation, Alexander’s staining, microscopic observation, and the ATP quantification were used to functionally validate the role of miR9569 in pollen development. The pollen of OE-miR9569 *Arabidopsis thaliana* at anthesis was significantly different from that of the wild type. Anther viability and ATP content were significantly lower than that of wild-type *Arabidopsis thaliana*. The ROS content was significantly higher than that of the wild type, as observed by confocal microscopy, and defective exine patterning were produced, as observed by scanning electron microscopy.

In summary, the mitochondrial gene *orf138* mutation regulates the downregulation of miR9569, which targets the ATP related gene, *AHA6*, blocking downstream H^+^-ATPases. The lack of energy leads to the obstruction of the normal formation of the pollen wall and ultimately causes pollen abortion.

## Figures and Tables

**Table 1:** Statistics of transcriptome sequencing data evaluation of anther samples from two lines.

Sample ID	Samples	Clean Reads	GC (%)	Q30 (%)	Mapped Reads
S07	ML1-1	68,768,532	43.85	95.99	51,673,715 (75.14%)
S08	ML1-2	85,298,784	44.47	96.03	64,307,383 (75.39%)
S09	ML1-3	78,197,288	45.19	96.02	60,180,391 (76.96%)
	ML1-average	77,421,535	44.50	96.01	58,720,496 (75.83%)
S10	CMS1-1	73,943,440	43.46	95.80	52,303,161 (70.73%)
S11	CMS1-2	80,664,306	45.57	96.16	58,978,727 (73.12%)
S12	CMS1-3	74,097,366	45.38	95.99	53,966,421 (72.83%)
	CMS1-average	76,235,037	44.80	95.98	55,082,768 (72.23%)
S01	ML2-1	83,770,774	45.19	95.64	77,887,958 (89.76%)
S02	ML2-2	77,257,468	44.90	96.01	69,431,461 (89.87%)
S03	ML2-3	75,009,460	44.93	95.74	64,617,111 (86.15%)
	ML2-average	78,679,234	45.01	95.80	70,645,510 (88.59%)
S04	CMS2-1	77,760,428	45.07	95.99	60,702,156 (78.06%)
S05	CMS2-2	69,712,214	44.69	96.06	52,983,713 (76.00%)
S06	CMS2-3	80,281,016	45.42	95.96	63,100,530 (78.60%)
	CMS2-average	75,917,886	45.06	96.00	58,928,800 (77.55%)

**Table 2:** SNP (single nucleotide polymorphism) locus statistics of anther samples from two lines.

Sample ID	Sample	SNP Number	Genic SNPs	Transition	Transversion	Heterozygosity
S07	ML1-1	216,700	170,197	57.24%	42.76%	29.14%
S08	ML1-2	237,398	184,124	57.04%	42.96%	29.68%
S09	ML1-3	208,136	166,036	57.31%	42.69%	29.24%
	ML1-average	220,745	173,452	57.20%	42.80%	29.35%
S10	CMS1-1	227,465	179,713	57.09%	42.91%	45.29%
S11	CMS1-2	230,515	182,973	57.25%	42.75%	44.86%
S12	CMS1-3	225,547	179,233	57.32%	42.68%	44.34%
	CMS1-average	227,842	180,640	57.22%	42.78%	44.83%
S01	ML2-1	176,283	140,122	57.69%	42.31%	28.57%
S02	ML2-2	172,702	137,363	57.68%	42.32%	28.27%
S03	ML2-3	173,190	138,322	57.66%	42.34%	28.53%
	ML2-average	174,058	138,602	57.68%	42.32%	28.46%
S04	CMS2-1	199,387	157,597	57.35%	42.65%	39.67%
S05	CMS2-2	188,110	148,740	57.42%	42.58%	38.77%
S06	CMS2-3	197,367	156,199	57.38%	42.62%	39.18%
	CMS2-average	194,955	154,179	57.38%	42.62%	39.21%

**Table 3:** Sample miRNA sequencing data assessment statistics.

Samples	Sample ID	Raw_Reads	Containing ‘N’ Reads	Length < 18	Length > 30	Clean Reads	Q30 (%)
ML1	S07	15,893,277	144	3,955,883	1,122,051	10,815,199	96.87
ML1	S08	20,103,763	78	5,518,188	1,542,877	13,042,620	96.11
ML1	S09	19,280,153	70	3,545,553	2,318,058	13,416,472	96.73
CMS1	S10	15,264,422	93	2,935,708	907,131	11,421,490	96.70
CMS1	S11	41,575,041	67	22,906,812	1,512,066	17,156,096	96.59
CMS1	S12	15,261,148	116	1,457,723	2,035,911	11,767,398	96.36
ML2	S01	13,467,807	42	2,319,292	911,338	10,237,135	97.48
ML2	S02	17,142,925	41	4,425,026	868,529	11,849,329	97.04
ML2	S03	13,400,654	40	850,586	1,322,760	11,227,268	97.27
CMS2	S04	20,246,038	111	9,026,132	1,174,822	10,044,973	96.86
CMS2	S05	20,265,519	111	7,341,508	1,495,005	11,428,895	96.93
CMS2	S06	21,509,385	124	9,318,264	960,142	11,230,855	96.88

**Table 4:** Sequence comparison results of the sample sequence data with the reference genome.

Type	Tag Number	Percent
Total	6,944,263	100%
Mapped	4,618,210	66.50%
Perfect map	3,657,820	79.20%
Imperfect map	960,390	20.80%
Unmapped	2,326,053	32.50%

## Data Availability

The original contributions presented in the study are publicly available. The transcriptome data can be found here: National Center for Biotechnology Information (NCBI) BioProject database under accession number PRJNA657160. The miRNAs and degradome data can be found here: China National GeneBank DataBase (CNGBdb) under accession numbers CNP0006167 and CNP0006176.

## Supplementary Materials

The following supporting information can be downloaded at: https://www.mdpi.com/article/10.3390/plants14162604/s1, Supplementary Material SA. Figure S1. Small RNA sequence length statistics; Figure S2. miR9569 shear target gene *Bra013168* (*AHA6*). Supplementary Material SB. Differentially expressed miRNAs targeting differentially expressed mRNAs during different developmental periods in pollen of Ogura CMS and maintained lines of Chinese cabbage. Supplementary Material SC. miR9569 matrices sequences and precursor sequences and other related information; qRT-PCR related primer information. Supplementary Material SD. Bioinformatics analysis of miR9569. Figure S1. miR9569 target gene structure analysis and target location prediction; Figure S2. Chromosomal localization of pre-miR9569 and its target genes; lanes 1, 2, 3, and 4: maintenance (Y231-330) and sterile (Tyms) lines were used as background genes, respectively; Figure S3. Secondary structure prediction of miR9569 precursor; Figure S4. Fragment of miR9569 precursor sequence obtained; Figure S5. miR9569 mature sequence base conservation analysis. Supplementary Material SE. The information of OE-miR9569 vector. Figure S1. OE-miR9569 recombinant plasmid verification gel electrophoresis map; Figure S2. A schematic diagram of the construction of the OE-miR9569 vector; Figure S3. OE-miR9569 colony PCR gel electrophoresis.
