# Supplementary material for: bra-miR9569 Targets the BrAHA6 Gene to Negatively Regulate H+-ATPases, Affecting Pollen Fertility in Chinese Cabbage (Brassica rapa L. ssp. pekinensis)
Source: Plants (Basel). 2025 Aug 21;14(16):2604. doi: 10.3390/plants14162604 (PMC12389279; doi:10.3390/plants14162604)
Supplement: Supplementary file 1 [file plants-14-02604-s001.zip › Supplementary Material SB.pdf]

Table S1. Differentially expressed miRNAs targeting differentially expressed mRNAs during different developmental periods in pollen of Ogura CMS and maintained lines of cabbage

miRNA name	Target gene	NR_annotation
miR158	*Bra027656*	restorer-of-fertility
	Bra000531	Predicted protein
miR159	*Bra022962*	Hypothetical protein ARALYDRAFT_902483
	Bra021791	Putative transcription factor MYB101
	Bra026958	hypothetical protein
	Bra031731	hypothetical protein
miR5654	*Bra026836*	pentatricopeptide repeat-containing protein(PPR)
	Bra016758	DEAD-box ATP-dependent RNA helicase 47(mitochondrial)
	Bra026884	hypothetical protein
miR860	*Bra014184*	Cytochrome c biogenesis protein family（CCS1）
	Bra030219	IAA8
	Bra008407	Metacaspase 5
miR9569	*Bra012414*	Betv I allergen family protein
	Bra013168	H^+-ATPase 6（AHA6）
	Bra012398	Betv I allergen family protein
novel_mir_354	*Bra032694*	ATP binding kinase protein kinase
novel_mir_403	*Bra024504*	cyclin 2a protein [*Arabidopsis thaliana*]
	Bra037265	cyclin 2a protein [*Arabidopsis thaliana*]
novel_mir_448	*Bra007991*	Sucrose transporter(SUC1)
	Bra016103	Sucrose transporter(SUC1)
novel_mir_51	*Bra035074*	V-type proton ATPase catalytic subunit A-like
novel_mir_95	*Bra034358*	S-adenosyl-L-methionine-dependent methyltransferases superfamily protein
