# Supplementary material for: bra-miR9569 Targets the BrAHA6 Gene to Negatively Regulate H+-ATPases, Affecting Pollen Fertility in Chinese Cabbage (Brassica rapa L. ssp. pekinensis)
Source: Plants (Basel). 2025 Aug 21;14(16):2604. doi: 10.3390/plants14162604 (PMC12389279; doi:10.3390/plants14162604)
Supplement: Supplementary file 1 [file plants-14-02604-s001.zip › Supplementary Material SD.pdf]

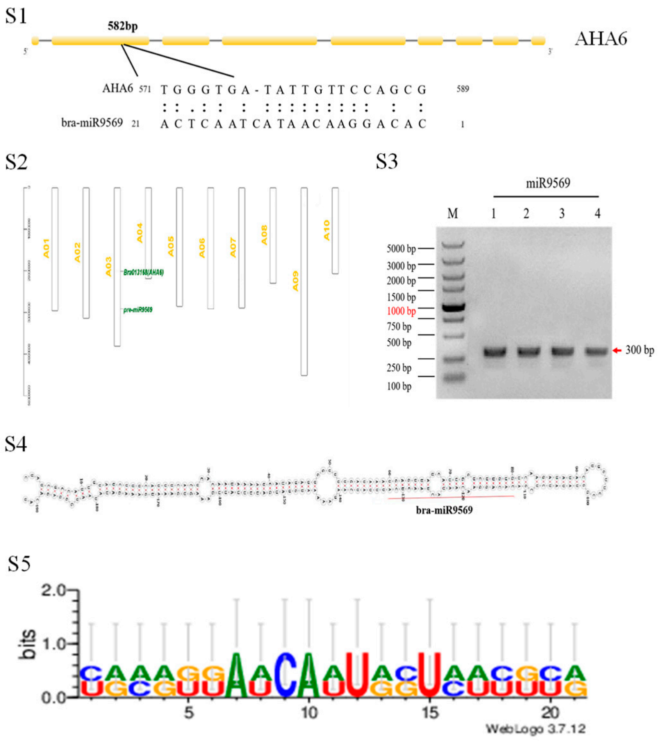

Fig S1. Structural analysis of miR9569 target genes and prediction of target sites;

Fig S2. Chromosomal localization of pre-miR9569 and its target genes;

Fig S3. PCR amplification of pre-miR9569. Bands 1, 2, 3, and 4: using the maintenance strain (Y231-330) and sterile strain (Tyms) as background genes, respectively;

Fig S4. Secondary structure prediction of the miR9569 precursor molecule, with the red line marking the mature sequence of miR9569;

Fig S5. Base conservation analysis of the mature sequence of miR9569.
