# Supplementary material for: bra-miR9569 Targets the BrAHA6 Gene to Negatively Regulate H+-ATPases, Affecting Pollen Fertility in Chinese Cabbage (Brassica rapa L. ssp. pekinensis)
Source: Plants (Basel). 2025 Aug 21;14(16):2604. doi: 10.3390/plants14162604 (PMC12389279; doi:10.3390/plants14162604)
Supplement: Supplementary file 1 [file plants-14-02604-s001.zip › Supplementary Material SE.pdf]

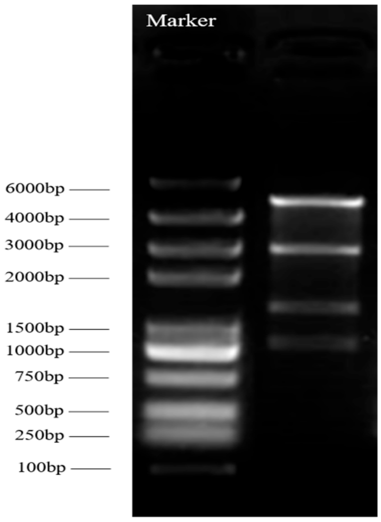

Fig S1. OE-miR9569 recombinant plasmid verification gel electrophoresis map

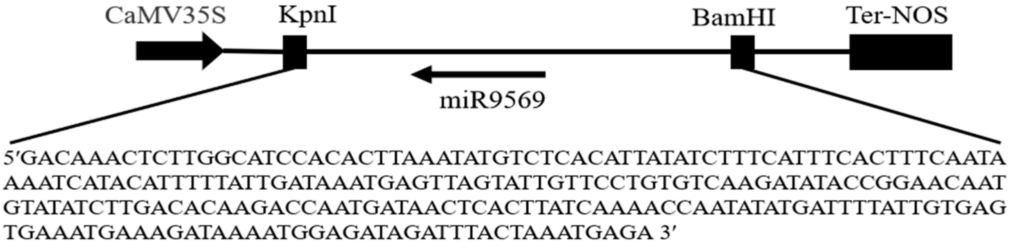

Fig S2. A schematic diagram of the construction of the OE-miR9569 vector

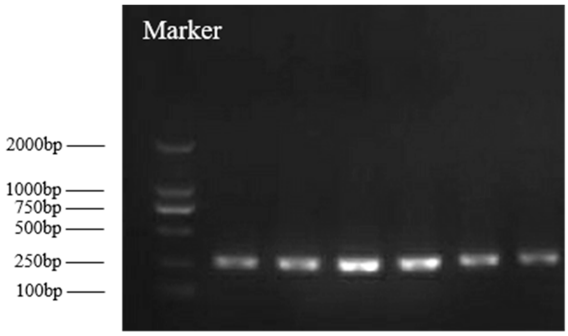

Fig S3. OE-miR9569 colony PCR gel electrophoresis
